# Magnolol inhibits venous remodeling in mice

**DOI:** 10.1038/s41598-017-17910-0

**Published:** 2017-12-19

**Authors:** Hanna Kuk, Caroline Arnold, Ralph Meyer, Markus Hecker, Thomas Korff

**Affiliations:** Institute of Physiology and Pathophysiology, Division of Cardiovascular Physiology, Heidelberg University, Heidelberg, Germany

## Abstract

Due to gravity the venous vasculature in the lower extremities is exposed to elevated pressure levels which may be amplified by obesity or pregnancy. As a consequence, venules dilate and may be slowly transformed into varicose or spider veins. In fact, chronically elevated venous pressure was sufficient to cause the corkscrew-like enlargement of superficial veins in mice. We hypothesized that biomechanical activation of endothelial cells contributes to this process and investigated the inhibitory capacity of Magnolol in this context – a natural compound that features multiple properties counteracting cellular stress. While Magnolol did not influence endothelial capillary sprout formation, it interfered with proliferation, ERK1/2 activity, gelatinase activity as well as baseline production of reactive oxygen species in these cells or murine veins. The anti-oxidative and anti-proliferative capacity of Magnolol was mediated through stimulation of heme oxygenase-1 expression. Finally, local transdermal application of Magnolol attenuated pressure-mediated development of varicose/spider veins in mice and was accompanied by the absence of proliferating and MMP-2 positive endothelial cells. Collectively, our data identified Magnolol as a potent inhibitor of biomechanically evoked endothelial cell activity during pressure-mediated venous remodeling processes which contribute to the development of varicose and spider veins.

## Introduction

Venous diseases are not only associated with thrombosis but also include the slowly progressive functional and structural impairment of the venous wall which may ultimately lead to the development of varicose/spider veins and chronic venous insufficiency. In fact, these diseases are frequently seen in North America and Western Europe, with over 20% prevalence in the adult western population and an estimated 25 million of Americans affected by varicose veins each year^1,2^. Varicose veins are classified as enlarged and tortuous superficial veins in the subcutaneous tissue, 3 mm in diameter or larger^1,3,4^. They are commonly associated with swollen, painful legs which may potentially develop complications including skin ulcers, superficial thrombophlebitis, bleeding and inability to stand or to walk^4,5^.

A number of risk factors have been associated with the development of varicose/spider veins, including prolonged standing, being overweight, pregnancy, prolonged sitting with legs crossed, wearing tight undergarments or clothes, history of blood clots (impaired venous return) which directly or indirectly support a rise in the venous filling pressure in lower extremities^6,7^. Consequently, it has been suggested that the resultant biomechanical (over)load, along with valvular dysfunction, may chronically elevate the wall stress in the associated venous network to drive its remodeling and dilatation^6–8^. This biomechanical stimulus sufficient for varicose vein development and progression may also pose as a predominant mechanism underlying spider vein formation when venous hypertension spreads to the smaller and superficial veins. In contrast to larger (varicose) veins, remodeling of smaller superficial blood vessels may be primarily driven by activation of endothelial cells lining the luminal vessel wall, given a thinner smooth muscle cell layer. Therefore, counteracting the biomechanically evoked activation of endothelial cells may effectively interfere with the remodeling of smaller veins.

To this end, we utilized Magnolol: a polyphenolic organic compound derived from the bark of magnolia tree including Magnolia officinalis, M. obovate and other species of the Magnoliaceae family^9^. Magnolol was reported previously to affect vascular remodeling processes associated with neointima formation and atherosclerosis and has been reviewed for its anti-ageing and protective effects on the skin^10–12^. In contrast to the numerous studies also involving vascular smooth muscle cell biology, effects of Magnolol on vascular endothelial cells remain largely unknown. A study in 2013 has reported that Magnolol inhibits vascular endothelial cell growth factor (VEGF) induced human umbilical vein endothelial cell (HUVEC) proliferation, chemotactic motility and tube formation in culture, primarily mediated via rat sarcoma- (Ras), extracellular signal–regulated kinase- (ERK), Phosphatidylinositol-4,5-bisphosphate 3/protein kinase B- (PI3K/Akt) and p38-associated pathways^13^. This study was intended to characterize the impact of Magnolol on the functional properties of biomechanically stimulated venous endothelial cells *in vitro* and *in vivo*.

## Materials and Methods

### Antibodies and reagents

The anti-phospho-p44/42 MAPK and anti-p44/42 MAPK rabbit monoclonal antibodies were obtained from Cell Signaling Technology (Danvers, Massachusetts; USA; 4370S and 4695). The anti-Ki67 antibody was obtained from abcam (Cambridge, UK; ab16667), the anti-MMP-2 (matrix metalloproteinase-2) rabbit polyclonal antibody was obtained from Dianova (Hamburg, Germany; DLN-012481), the anti-HO-1 (heme oxygenase-1) mouse monoclonal antibody was obtained from BD Biosciences (*San Jose*, *California*; cat. No: 610712) Magnolol was obtained from Sigma-Aldrich (St. Louis, Missouri; M3445). Tin protoporphyrin IX dichloride (SnPPIX) was obtained from Santa Cruz (Dallas, Texas; SC-203452).

### HUVEC cell culture

Human umbilical vein endothelial cells (HUVECs) were isolated as per approval from the Local Ethical Committee (document number 336/2005, Heidelberg Germany) and conformed to the principles outlined in the Declaration of Helsinki (1997). Parental consent was obtained for isolation of HUVECs from the umbilical cords of the newborns. Umbilical veins were flushed with Hank’s buffer solution to remove residual blood. The veins were filled with 10 ml of dispase solution (3.1 g/l) and incubated for 30 minutes at 37 °C. Veins were then flushed with 40 ml of M199 medium (Gibco, ThermoFisher Scientific, Massachusetts, USA) resulting in a cell-containing media suspension. Both M199 and dispase solutions were centrifuged at 1000 rpm for 5 min and the cell pellet was re-suspended in M199 media (Sigma-Aldrich, Germany) supplemented with endothelial cell growth supplement (PromoCell, Germany), 5% FBS (Gibco, ThermoFisher Scientific, Massachusetts, USA), 50 U/ml penicillin, 50 μg/ml streptomycin and 0.25 μg/ml Fungizone® antimycotic (Gibco, ThermoFisher Scientific, Massachusetts, USA). The endothelial phenotype of these cells was confirmed by positive immunofluorescence for the endothelial marker CD31 and assessment of a cobble-stone like morphology. The cells were routinely cultured on standard plates pre-coated with 2% (w/v) gelatinand transferred on surfaces pre-coated (1 hour at 37 °C) with Geltrex® (basement membrane surrogate, 1:10 in cell media; ThermoFisher Scientific, Massachusetts, USA) prior to any experiment. Only cells subcultured up to passage 4 were utilized for all subsequent experiments.

### Western Blot

HUVEC monolayers were lysed and the 20 µg of denatured protein samples were loaded onto 10% SDS (sodium dodecyl sulfate) gels and separated by gel electrophoresis followed by transfer to a nitrocellulose membrane. The membranes were blocked and incubated overnight at 4 °C in the presence of a primary antibody (dilution as per manufacturer instruction). The following day, membranes were incubated with the secondary horseradish peroxidase- (HRP) conjugated antibody (dilution as suggested by the manufacturer) and developed with LuminataTM Forte western HRP substrate (Millipore, Massachusetts, USA) according to the manufacturer´s instruction using the Image Quant TM LAS 4000 mini machine (GE Healthcare). Protein band signal intensity was quantified using ImageJ (NIH, Maryland, USA) software.

### Immunofluorescence staining

HUVEC cells were fixed with methanol for 15 minutes at 4 °C, air- dried and blocked with 1% bovine serum albumin in phosphate buffered saline (BSA/PBS) buffer for 30 min. The cells were incubated at 4 °C overnight with the corresponding primary antibodies, washed and labelled with the appropriate fluorophore -conjugated secondary antibodies (Dianova) for 1 hour at room temperature. Nuclei were visualized by counterstaining with DAPI (2 μg/mL, diluted in PBS) for 10 minutes and cells were mounted with Mowiol (Fluka, Missouri, USA). Determination of gelatinase activity was performed by incubating methanol-fixed cells with fluorescein-conjugated DQ-gelatin (EnzChek gelatinase assay kit; ThermoFisher Scientific, Massachusetts, USA) for 1 hour at 37 °C as per manufacturer’s instructions. Immunofluorescence staining intensity was captured and quantified by using the Cell^R software (Olympus, Hamburg, Germany) analyzing at 4–5 different regions of the specimen per experimental group. Exposure times during digital imaging were kept constant. TissueGnostics/TissueQuest microscopy (Axiovert 200 M, Zeiss, Germany) and software were utilized to simultaneously correlate the number of Ki67 positive cells with the Ki67 signal intensity in a scatterplot graph format.

#### Detection of reactive oxygen species (ROS)

Levels of Reactive oxygen species (ROS) in HUVEC l cultures were assessed essentially as described^14,15^. Briefly, Mangolol or solvent- (dimethyl sulfoxide, DMSO) treated HUVECs were subject to biomechanical stretch for 6 or 24 hours followed by incubation with Dihydroethidium (DHE; 10 µM in DPBS; Sigma-Aldrich, Missouri, USA) or 5(6)-Carboxy-2′,7′-dichlorofluorescein diacetate (DCFDF; 10 µM in DPBS; Sigma-Aldrich, Missouri, USA) fluorescence ROS detection dyes for 30 min at 37 °C and 5% CO_2_. The cells were washed and the levels of fluorescence intensity determined as described above.

### Profiler Arrays

Profiling of protein phosphorylation was performed by utilizing a corresponding array (ARY003B; R&D, Minnesota, USA) as per manufacturer’s instruction. Briefly, cell lysates (175 µg total protein) were incubated at 4 °C overnight with the nitrocellulose membranes where capture and control antibodies have been spotted in duplicates. Membranes were washed and incubated with a cocktail of biotinylated detection antibodies for 2 hours at room temperature. Streptavidin-HRP reagent was added to the washed membranes for an additional 30 min interval after which time the membranes were developed with chemiluminescent detection substrate using Image Quant TM LAS 4000 mini machine (GE Healthcare). The signal produced at each capture spot was quantified using ImageJ software and corresponds to the amount of phosphorylated protein bound. The relative protein level of human proteases was assessed using a Human Protease Array Kit (ARY021B; R&D, Minnesota, USA) as per manufacturer’s instruction with the signal captured and quantified essentially as described above.

### Toxicity Assay

Viability of HUVECs cultured with increasing Magnolol concentration for 6 and 24 hours was assessed with a PrestoBlue cell Viability Reagent (Invitrogen, Massachusetts,U.S.) as per manufacturer’s instructions. Fluorescence readings were taken at 544 nm/590 nm excitation and emission wavelength respectively (Fluoroskan Ascent, fluorescence reader, ThermoFisher Scientific, Massachusetts, USA) which correspond directly to the amount of viable cells.

### Angiogenesis Assay

Endothelial cell spheroids of defined cell number were generated as described previously^16^. In brief, HUVECs were suspended in the culture medium containing 0.25% (w/v) methylcellulose and seeded into non-adhesive round bottom 96-well plates (Greiner, Germany) overnight allowing for the formation of a single, well-defined a round spheroid in each of the tissue culture wells (500 cells per spheroid were seeded). Spheroid were collected the next day and embedded into liquid collagen gels which were allowed to polymerize. Magnolol or DMSO control were dissolved in endothelial cell basal media (Promocell, Heidelberg, Germany) containing VEGF (25 ng/mL; PromoKine, Heidelberg, Germany) and pipetted onto the gels. The gels were incubated at 37 °C, 5% CO_2_ for 24 hours and the angiogenesis was quantitated by measuring the length and the number of the sprouts (calculated as cumulative sprout length) that had grown out of each spheroid obtained by microscopy imaging using Cell digital software (Olympus, Hamburg, Germany).

### Proliferation cell count

HUVECs were treated with Magnolol (10 µM, 40 µM) at ~30% confluency and allowed to grow for an additional 24 hours. Light microscopy images from the same areas of the well were obtained immediately after pre-treatment as well as 24 hours and the total number of cells in each field of view was quantified with the help of ImageJ software (NIH, Maryland, USA). The cell number was converted into doubling time using the following formula:

$$\mathrm{Doubing}\,\mathrm{time}\,=\,\frac{\mathrm{time}\,\mathrm{in}\,\mathrm{culture}\ast \,\mathrm{Log}\,(2)}{\mathrm{Log}\,(\mathrm{final}\,\mathrm{cell}\,\#\,)-\,\mathrm{Log}\,(\mathrm{initial}\,\mathrm{cell}\,\#)\,}$$

### Animal models

All animal studies were approved by the Regional Council Karlsruhe and carried out in accordance with the Guide for the Care and Use of Laboratory Animals published by the US National Institutes of Health (NIH Publication No. 85-23, revised 1996). Ligation of mouse auricle veins was performed as described earlier^17,18^. In brief, male NMRI mice (at least 12 weeks of age) were anesthetized with isoflurane (Baxter, Illinois, USA) and one of the three first order veins was ligated using a surgical thread (silk, 7.0, Ethicon, Norderstedt). Due to the highly collateralized auricle vasculature, this procedure does not lead to ischemia or necrosis. Remodeling of collateral veins was documented on a daily basis by using a high-resolution digital camera (Digital IXUS 8515, Canon, Tokio, Japan) and the PeriCam PSI blood perfusion imaging system (Perimed, Stockholm, Sweden) to control the local perfusion conditions. High resolution images obtained with a digital camera adjusted to a dissecting microscope (Microscope Wild, Heerbrugg; Leica, Wetzlar, Germany) with a full view of the murine auricle, essentially as described by North and Sanders^17^. Images were then morphometrically analyzed using the software Image J (NIH, Maryland, USA) by measuring across the diameter of the remodeling vessels. A cetearyl alcohol-based lubricious cream formulation (Unguentum emulsificans aquosum; Caesar & Loretz GmbH; Hilden, Germany) containing Magnolol (20 μg/ear, roughly matches an effective concentration of 20–40 µM Magnolol considering tissue/cartilage volume, penetration time and drainage) or DMSO vehicle control (1% by weight) was transdermally administered to the auricles of mice one day prior as well as after ligation surgery (every day or every second day in the follow up study). Four days upon ligation, mice were sacrificed and perfused with Ringer solution and zinc-fixative. Mouse auricles were dissected and processed for paraffin embedding and histological examination.

### Perfusion of isolated mouse veins

Animals were sacrificed and the facial and saphenous veins were extracted and inserted into a perfusion chamber (Culture Myograph, DMT, Copenhagen, Denmark). The chambers were placed in an incubator at 37 °C and 5% CO_2_, and the veins were continuously perfused for 18–24 hours at a transmural pressure of 4 mm Hg or 16 mmHg in Panserine 401 media (PAN-Biotech, Aidenbach, Germany) in the presence of Magnolol (40 µM) or the equivalent volume of DMSO vehicle control. After perfusion, vessel segments were fixed in Dent’s fixative (4 °C for 24 hours) and subsequently processed for whole-mount immunofluorescence analyses.

### Mouse saphenous vein ligation

Male NMRI mice (12 weeks old) were anesthetized with isoflurane. Underlying vasculature of a hind limb was surgically exposed and saphenous vein was ligated at a region distal from its origin. Sham mice did not undergo vein ligation. Seven days after the surgery the mice were euthanized by CO_2_ inhalation and cervical dislocation. The left ventricle of the heart was cannulated and perfused for 2 min at 90–100 mm Hg with Ringer solution containing 0.1% adenosine and 0.05% BSA (w/v) at 37 °C followed by retrograde perfusion (through the right ventricle) with a colored pigment (16% of HKS® Designers’ Gouache (Schmincke) in zink fixative). The pigment cannot pass the capillary system, allowing for the morphological assessment of venules and small veins while fixed at full (pressure-induced) dilation. The hindlimbs were then dehydrated using a series of alcohol and isopropanol and finally incubated in a mixture of benzyl alcohol and benzyl benzoate (1:1, v/v) having the same refractive index of the tissue for at least 18 hours. This procedure induces transparency of the tissue and allows detailed analysis of the pigment-loaded and fully dilated venous system. The diameter of the veins in the lower limb was measured by using the morphological analysis software Cell^R from Olympus (Hamburg, Germany).

### Biomechanical stretch stimulation of HUVEC

To expose HUVECs to biomechanical stretch, cells were cultured on BioFlex Collagen type I 6-well plates (Flexcell, North Carolina, USA) pre-coated with Geltrex® (basement membrane surrogate, 1:10 in cell media; ThermoFisher Scientific, Massachusetts, USA) for 1 hour at 37 °C. One day prior to stretch, the endothelial cell supplement content of the media was reduced to half and diluted in the M199 media supplemented with 12.5% FCS (fetal calf serum), 25 U/ml penicillin, 25 μg/ml streptomycin and 0.125 μg/ml Fungizone® antimycotic. Cell monolayers were treated with Magnolol dissolved in DMSO at 10 µM or 40 µM final concentration or equivalent volume of DMSO vehicle control. Cyclic stretch was applied 1.5 hours later using a microprocessor controlled vacuum pump (FX-5000 FlexerCell strain unit, Flexcell, North Carolina, USA) with 15% cyclic elastomer elongation at frequency of 0.5 Hz. Cyclic, as opposed to static, elongation is needed to prevent the cells from evading the biomechanical stimulus through rearranging their focal contacts. Cells were stretched at 85–95% confluency.

### Whole-mount immunofluorescence

*Ex vivo* perfused vein segments were fixed in Dent’s fixative (80% methanol, 20% DMSO) for 30 min at room temperature. Vessels were then rehydrated in PBS with decreasing methanol content (75%, 50%, 25%) for 10 min, washed and incubated with a fluorescein-conjugated DQ-gelatin (EnzChek gelatinase assay kit; ThermoFisher Scientific, Massachusetts, USA) for 1 hour at 37 °C. To visualize HO-1 expression, vessels were incubated with a primary anti-HO-1 antibody overnight at 4 °C, washed in PBS and incubated with the corresponding secondary antibody overnight at 4 °C. 4′,6-Diamidin-2-phenylindol (DAPI, 2 μg/mL; Invitrogen, ThermoFisher Scientific, Massachusetts, USA) diluted in PBS was used for visualization of nuclei. Vessels were mounted longitudinally onto glass slides in the presence of Mowiol mounting media (Fluka, Sigma-Aldrich, Missouri, USA). Fluorescence signal intensity was captured and quantified using the Cell^R software (Olympus, Hamburg, Germany).

### Statistical analysis

Results are expressed as means ± SD, differences between 2 matched experimental groups were analyzed by unpaired Student’s *t*-test, with a probability value of p < 0.05 considered statistically significant (InStat 3.0 program; GraphPad, USA). Differences of one parameter among 3 or more experimental groups were analyzed by one-way ANOVA, followed by a Tukey-Kramer multiple comparisons test, with a probability value of p < 0.05 considered statistically significant. If not stated otherwise, bars represent the mean±SD of n independent experiments based on cells/veins from individual donors/mice.

## Results

### Increase in venous pressure is sufficient to drive the remodeling of superficial veins

**Figure 1:**
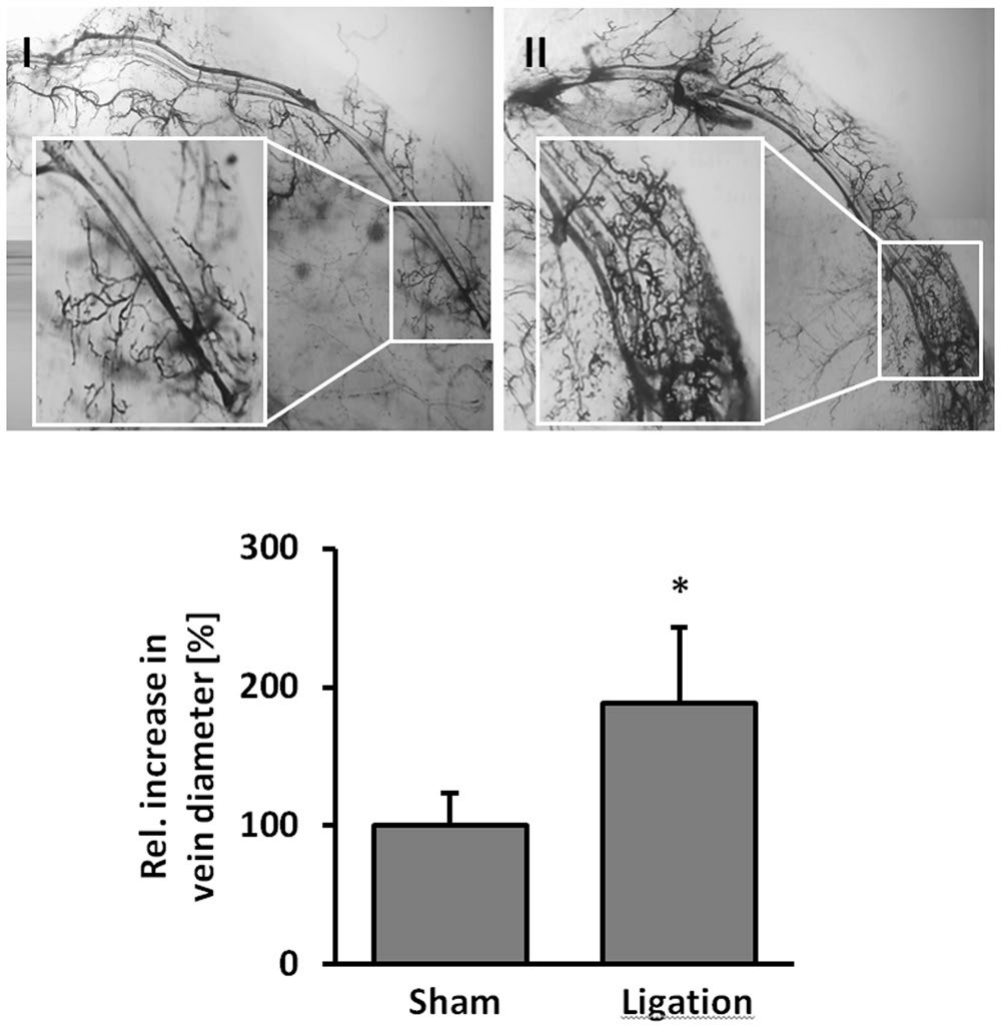
Morphometrical and functional analyses of superficial veins. Analysis of the venous circulation and structural changes of the superficial veins in the mouse hind limb 7 days after sham surgery ((**I)**, enlargement: normal-shaped veins connected to the saphenous vein) or following occlusion of a deep saphenous vein ((**II**), enlargement: dilation and tortuous architectural changes of the associated superficial veins 7 days after saphenous vein ligation, *p < 0.05 vs sham, n = 5). Retrograde perfusion of colored particles that cannot pass the capillary system was utilized in combination with microscopic high resolution digital imaging technique.

Superficial veins or venules are directly connected to the deeper venous plexus and may remodel to form bulged, tortuous and dilated (spider) veins in the superficial skin upon exposure to enhanced biomechanical load if the venous filling pressure increases. To investigate whether a chronic increase in the intraluminal pressure of the deeper veins is sufficient to induce remodeling/dilation of superficial veins, a (deep) saphenous vein was occluded in anesthetized mice to evoke venous hypertension (Fig. 1). This resulted in significant enlargement and corkscrew-like, tortuous architectural changes of the connected superficial veins 7 days after surgery (Fig. 1).

### Magnolol blocks proliferation and gelatinase activity but does not interfere with angiogenesis and baseline mitogen activated kinase (MAPK) activity of human endothelial cells

**Figure 2:**
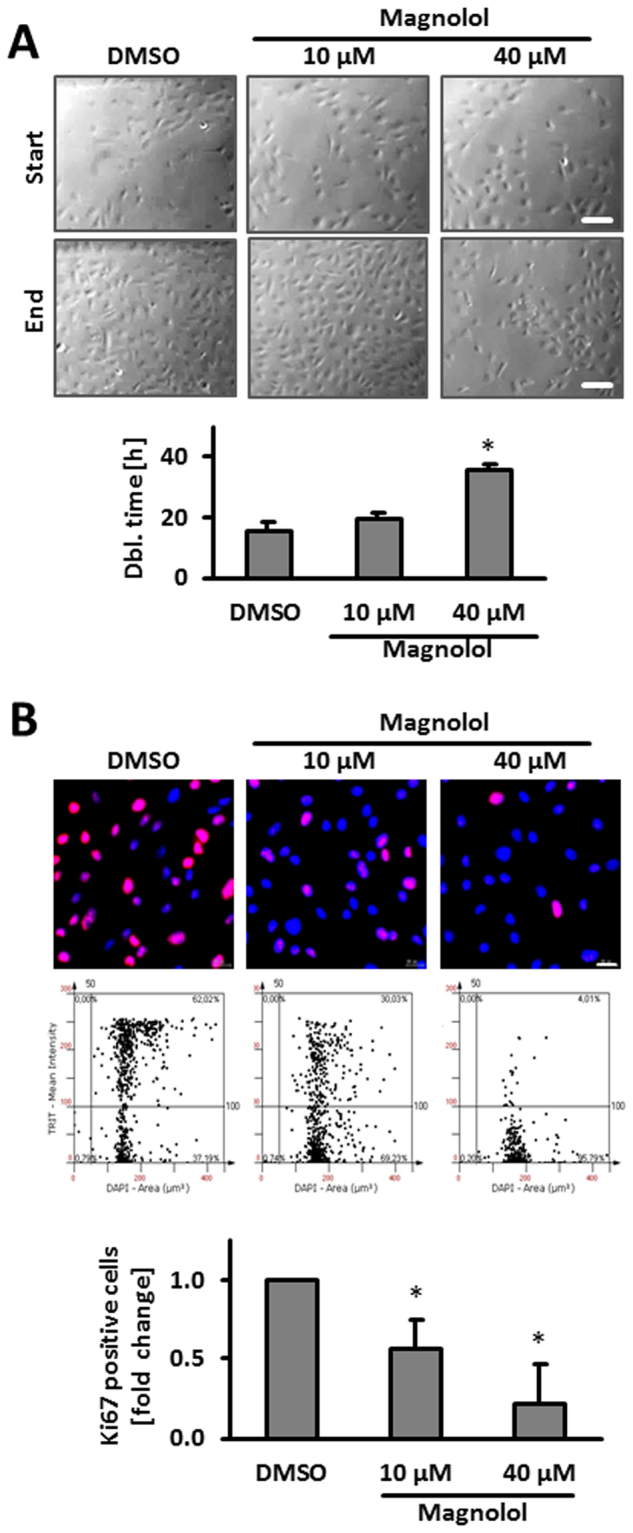
Magnolol inhibits HUVEC proliferation. (**A**) Proliferation cell count of HUVECs treated with Magnolol or DMSO. Doubling time was determined from a raw cell count within defined microscopic fields of view (MFV, *p < 0.05 vs. DMSO control, n = 3 (individual donors), scale bar: 200 μm). (**B**) Decrease in Ki67 staining intensity and number of Ki67-positive cells was observed upon 24 hours of treatment with Magnolol using automated immunofluorescence analysis (*p < 0.05 vs. control, n = 3–4, scale bar: 20 μm).

Considering the cell stress-protective features of Magnolol, we hypothesized that this compound is suitable to interfere with the biomechanical activation of endothelial cells and may thus effectively block venous remodeling. A prototypic response of endothelial cells during venous remodeling processes is enhanced proliferation^19,20^ which is a prerequisite for enlarging the inner surface of a vein thus enlarging its inner diameter. To estimate the putative inhibitory capacity of Magnolol in this context, we analyzed the impact of Magnolol on endothelial cell proliferation by assessing the doubling time of the cells in culture and by detecting the proliferation marker Ki67. Both approaches indicated a concentration dependent inhibitory effect of Magnolol (Fig. 2A,B). While this feature may interfere with maintenance of capillaries and angiogenesis and thus impair wound healing processes, we scrutinized the toxicity of Magnolol, its impact on angiogenesis as well as the baseline phosphorylation of proteins modulating endothelial cell homeostasis. Magnolol did not show any toxic effect on HUVECs or inhibited their capacity to form capillary sprouts in collagen gels (Supplemental Fig. 1A and B). Likewise, besides the activity of the protein kinase WNK1 (with no lysine (K) family kinase 1), Magnolol did not much influence the baseline phosphorylation of different kinases and its targets in cultured HUVECs (Supplemental Fig. 2).

**Figure 3:**
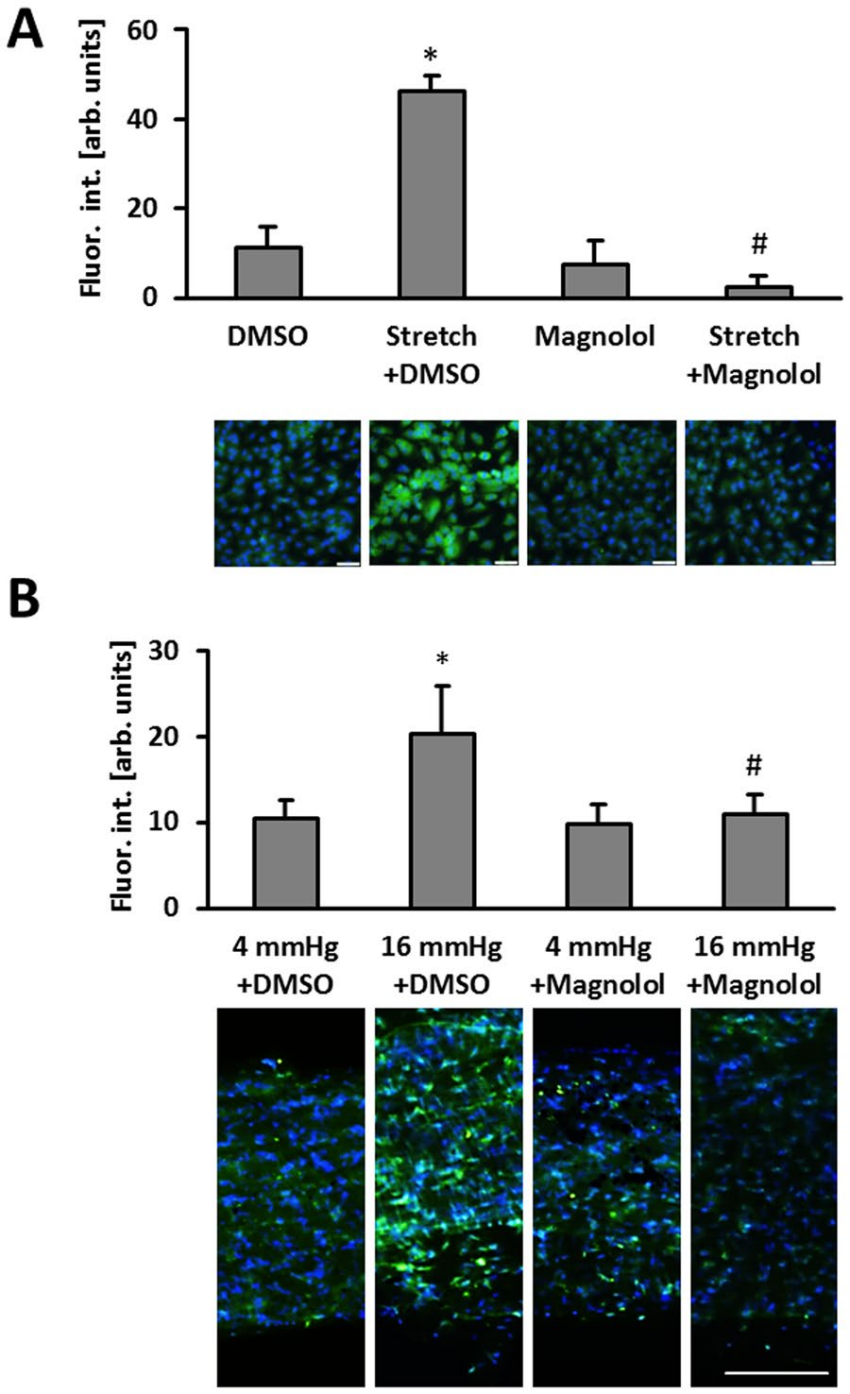
Magnolol inhibits HUVEC gelatinase activity. (**A**) HUVECs were exposed to biomechanical stretch for 24 hours with or without Magnolol pretreatment (40 µM). Gelatinase activity (green fluorescence) was determined by quantitative immunofluorescence analyses (*p < 0.05 static DMSO vs. stretch DMSO; ^#^p < 0.05 stretch DMSO vs. stretch Magnolol 40 µM; bars represent the mean ±SD of 4 MFV of 1 out of 6 experiments with comparable results; scale bars = 50 µm). (**B**) Mouse vein segments were exposed to basal (4 mmHg) and increased (16 mmHg) intraluminal pressures overnight with or without Magnolol (40 µM) pre-treatment. Gelatinase activity (green fluorescence) was assessed in a whole-mount staining procedure; nuclei were counterstained with DAPI (*p < 0.05 DMSO (4 mmHg) vs. DMSO (16 mmHg); ^#^p < 0.05 DMSO (16 mmHg) vs. 40 µM Magnolol (16 mmHg), n = 8–12).

As wall stress or stretch acts as a critical driving force of venous remodeling^7,18,21,22^ we aimed at evaluating the impact of Magnolol on prototypic stretch-induced responses of endothelial cells. To this end, we exemplarily assessed the changes in the phosphorylation of several protein kinases (MAPK, AMPK, FAK and Src kinases) in response to 15 minutes of experimental stretch (Supplemental Fig. 3A). At later points in time, most stretch-induced changes in the phosphorylation state of these protein kinases vanished (data not shown). As Magnolol appeared to preferentially dampen the initial stretch-mediated (activating) phosphorylation of MAP kinases, we verified this initial screening result by additional Western blot-based analyses (Supplemental Fig. 3B). Similarly, we investigated as to whether Magnolol influences the stretch-induced abundance of several proteases. While we did not observe any relevant changes in the level of proteases under these conditions (Supplemental Fig. 4), we revealed a Magnolol-mediated decrease in gelatinase activity of stretch-exposed endothelial cells (Fig. 3A). In line with the *in vitro* findings, gelatinase activity was also diminished upon Magnolol pre-treatment of murine veins exposed to elevated pressure levels (Fig. 3B).

### Magnolol lowers reactive oxygen species and promotes HO-1 levels

**Figure 4:**
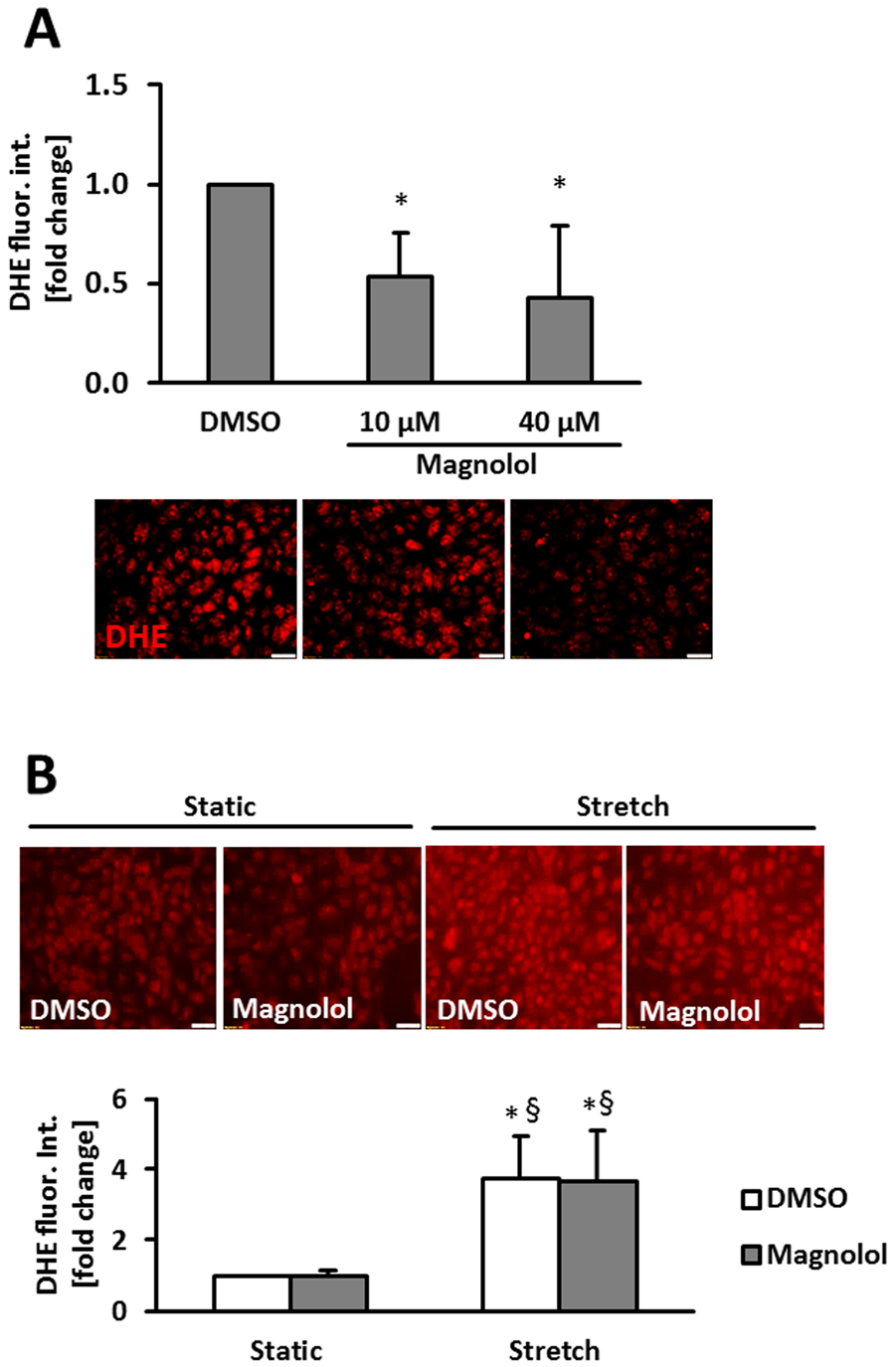
Magnolol attenuates endogenous ROS levels in HUVECs. (**A**) HUVECs were treated with Magnolol or DMSO vehicle control for 6 hours followed by DHE staining and quantitative immunofluorescence analyses to assess the levels of superoxide production. Microscopic fields of view with similar cell density levels were selected for analysis (*p < 0.05 DMSO vs Magnolol 40 µM, n = 6–7; scale bars = 50 µm). (**B**) HUVECs were treated with Magnolol or DMSO control vehicle and subject to 24 hours of biomechanical stretch. DHE staining was carried out to assess the levels of superoxide (**B**, *p < 0.05 vs. DMSO static, ^§^p < 0.05 vs. Magnolol static, n = 3; scale bar: 50 µm). The Magnolol-mediated decrease in DHE fluorescence under static conditions is not assessable in HUVECs cultured on Flexcell membranes which reduce the signal-to-noise ratio due to their autofluorescence.

Magnolol is known to act as an anti-oxidant compound^23,24^ which would at least partially explain the observed inhibitory effects. Thus, we investigated as to whether Magnolol would decrease the levels of ROS produced in endothelial cells exposed to biomechanical stretch^25,26^. While we observed a reduction in ROS levels upon Magnolol pre-treatment under static conditions (Fig. 4A, Supplemental Fig. 5A), ROS production in stretch-stimulated endothelial cells was not affected by the treatment with Magnolol (Fig. 4B, Supplemental Fig. 5B).

**Figure 5:**
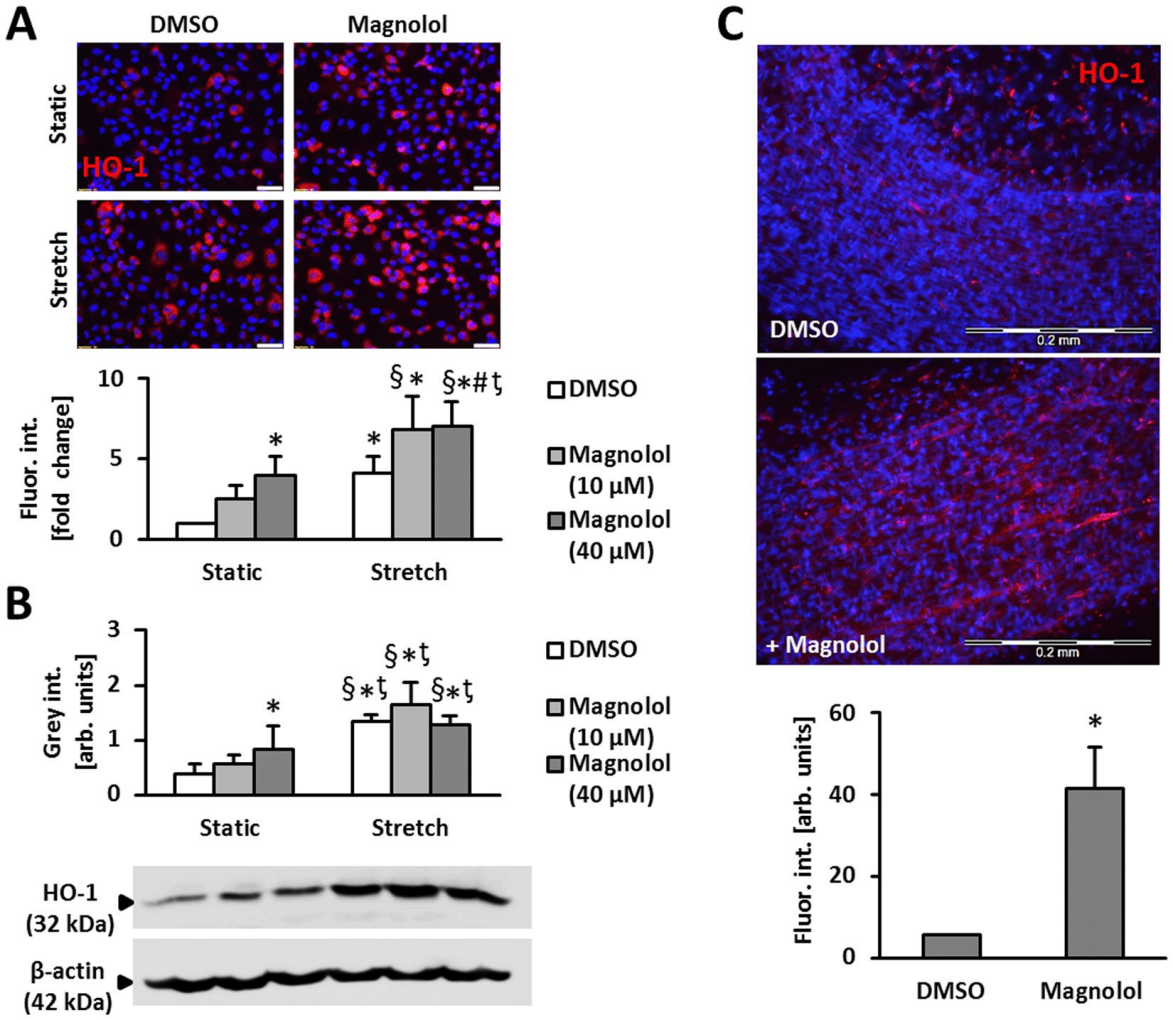
Magnolol simulates endogenous HO-1 levels in endothelial cells. (**A**) HUVECs were exposed to biomechanical stretch (15% cyclic elongation at 0.5 Hz) for 24 hours with or without Magnolol pretreatment (1.5 hours at 10 µM and 40 µM). Protein abundance of heme oxygenase-1 (HO-1) was determined by (**A**) quantitative immunofluorescence (*p < 0.05 vs. DMSO static; ^#^p < 0.05 vs. DMSO stretch; ^§^p < 0.05 vs. Magnolol 10 µM static; ^ƫ^p < 0.05 vs. Magnolol 40 µM static, n = 4; scale bars = 50 µm) or (**B**) Western blot analyses (*p < 0.05 vs DMSO static; ^§^p < 0.05 vs. Magnolol 10 µM static; ^ƫ^p < 0.05 vs. Magnolol 40 µM static, n = 6–8). (**C**) Mouse vein segments were pretreated with Magnolol (40 µM) or DMSO vehicle control for 24 hours. HO-1 levels (red fluorescence) were assessed by applying a whole-mount staining procedure in combination with confocal microscopy; nuclei were counterstained with DAPI. The HO-1 signal was attributed mostly to the EC layer (elongated cell shape, inner layer as evidenced by focal plane, *p < 0.05 vs. DMSO, n = 3, scale bars = 200 µm).

**Figure 6:**
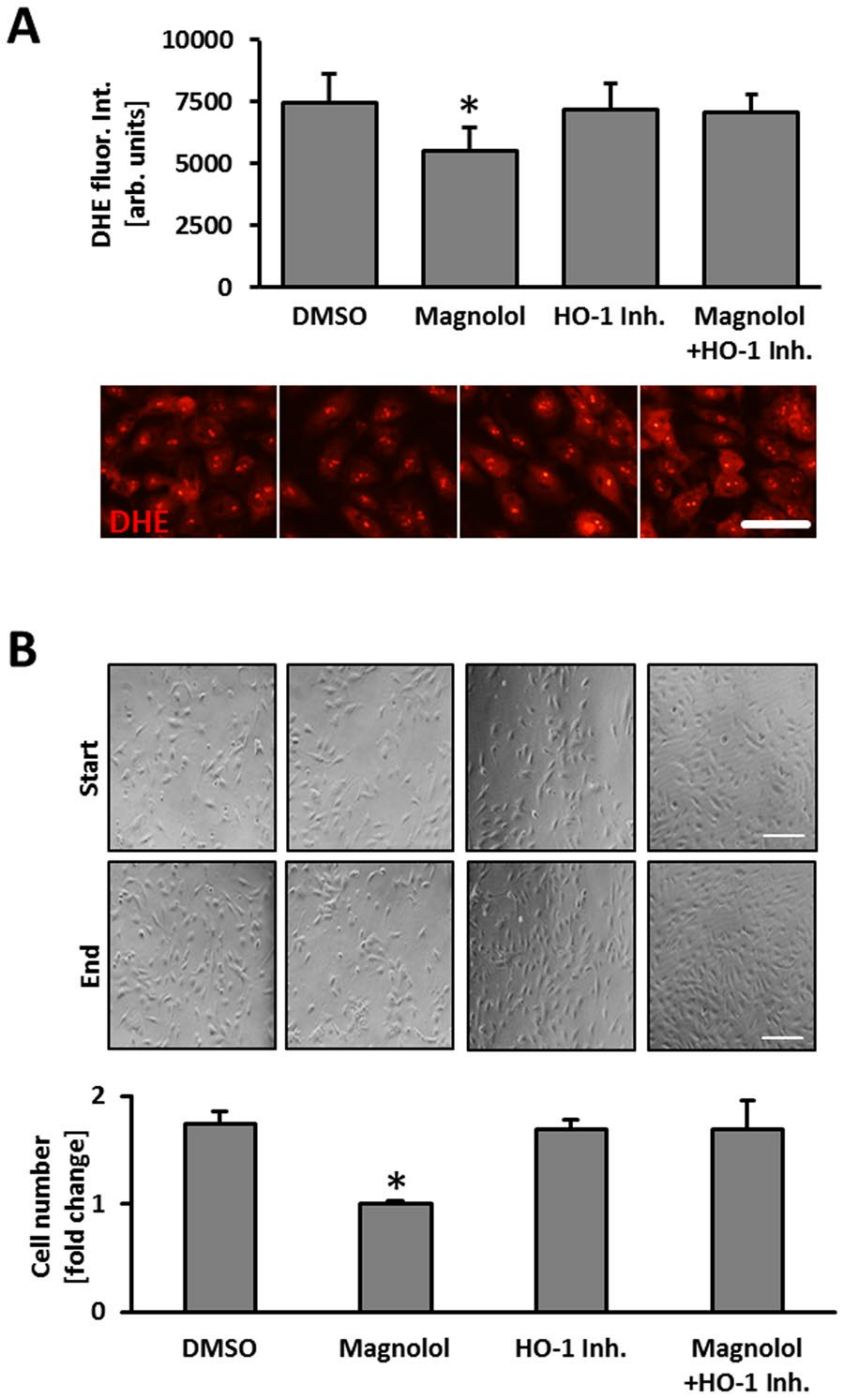
Inhibition of HO-1 blocks inhibitory effects of Magnolol. (**A**) HUVECs were treated with Magnolol (40 µM) in the presence of HO-1 inhibitor Tin protoporphyrin IX (HO1-Inh; 1 µM) or DMSO control for 6 hours followed by DHE staining and quantitative immunofluorescence analyses to assess the levels of superoxide production (DHE staining). Microscopic fields of view with similar cell density levels were selected for analysis (*p < 0.05 DMSO vs. Magnolol 40 µM, n = 6; scale bar = 50 µm). (**B**) Proliferation of HUVECs treated with Magnolol (40 µM) was determined in the presence of HO-1 inhibitor Tin protoporphyrin IX (HO-1 Inh) or DMSO control for 24 hours. Fold increase in the number of cells 24 hours later was assessed by raw cell count within defined MFV (*p < 0.05 vs. DMSO control, bars represent the mean ± SD of values obtained from 3 microscopic fields of view of 1 out of 2 experiments with comparable results, scale bar: 200 μm).

To delineate further putative effectors of Magnolol in stretch-exposed endothelial cells, we performed a microarray analysis. Based on HUVECs from 3 different donors, we identified heme oxygenase-1 (HO-1) as a target that may additionally limit ROS generation in stressed endothelial cells (Supplemental Fig. 6) which was partially confirmed on protein level (Fig. 5A–C). While Magnolol elevated the abundance of HO-1 under baseline conditions, only a slight additional increase was observed in total protein lysates from biomechanically stimulated HUVEC (Fig. 5B). The relevance of HO-1 for the inhibitory effects of Magnolol was thus investigated by blocking the activity of HO-1 in Magnolol-treated HUVEC. In fact, inhibition of HO-1 was sufficient to abrogate the Magnolol-mediated decline of the baseline ROS level as well as proliferation (Fig. 6A and B).

### Transdermal application of Magnolol prevents venous remodeling in the mouse auricle

**Figure 7:**
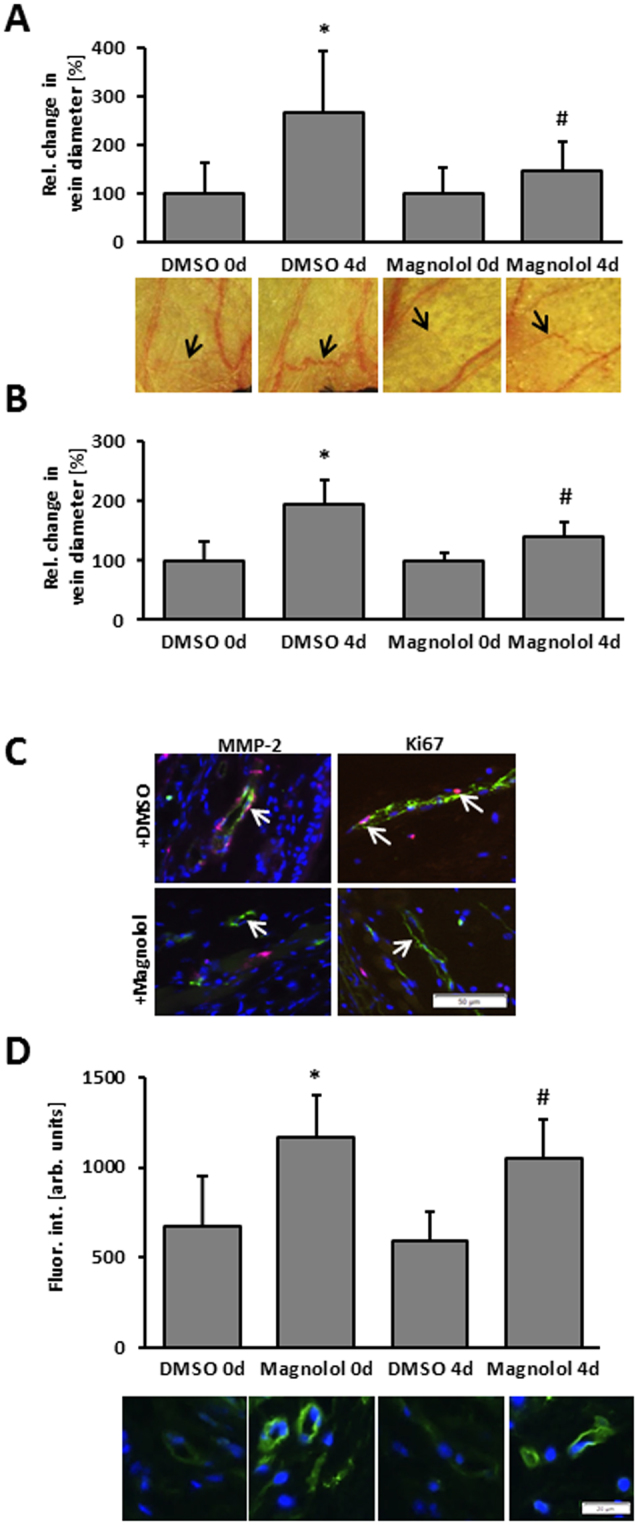
Magnolol attenuates spider vein-like remodeling in mouse auricles. (**A**) A first-order vein in the center of the mouse auricle was ligated to chronically raise the local pressure and induce the enlargement/remodeling of individual auricular venules (venous tributaries) connected to the corresponding venous network. This was quantitated by morphometrically analyzing high resolution digital images taken immediately after (0 d) and 4 days (4 d) after ligation. Magnolol or DMSO was applied as cream formulations to murine auricles (for details see materials and methods) on each of the 4 days subsequent ligation (**A**) or every other day (**B**). The original diameter of surveyed individual veins was set to 100% (*p < 0.05 DMSO 0 d vs. DMSO 4 d; ^#^p < 0.05 DMSO 4 d vs. Magnolol 4 d, n = 5–11 (**A**) and n = 6–8 (**B**)). (**C**) Representative images showing immunofluorescence staining of MMP2 and Ki67-positive cells along with an endothelial cell marker CD31 (green) in the remodeling veins from the mouse auricles. Nuclei were counterstained with DAPI (scale bar = 50 µm). (**D**) Immunofluorescence-based detection of HO-1 in mouse auricle veins treated with Magnolol or DMSO (vehicle control) in a cream formulation (nuclei were counterstained with DAPI, blue fluorescence). HO-1 specific staining intensity was determined by image analyses (*p < 0.05 vs. DMSO control, n = 6–9). No difference in the staining intensity was observed when comparing enlarged/remodeling or normal veins within treatment groups.

Considering the aforementioned findings, Magnolol may inhibit remodeling of small veins by boosting the abundance of HO-1 in endothelial cells prior to a biomechanical stimulus which may increase their initial resistance to ROS-mediated proliferative stimuli. On the other hand, Magnolol may directly inhibit gelatinase activity in biomechanically stimulated endothelial cells to limit structural alterations of the venous wall. To scrutinize the impact of Magnolol on venous remodeling *in vivo* a mouse model was applied that triggers remodeling of superficial veins over a course of up to seven days by locally increasing venous pressure^17,18^. This process involves the activation of the transcription factor activator protein 1 (AP-1), the production of metalloproteinases and proliferation of endothelial and smooth muscle cells. Transdermal application of Magnolol on a daily basis (Fig. 7A) or even every other day (Fig. 7B) significantly diminished the formation of dilated and tortuous veins in the auricle which was accompanied by a diminished endothelial proliferation and MMP-2 abundance (Fig. 7C). Although endothelial cells constitute the predominant cell type in these veins, MMP-2 may originate from both endothelial and smooth muscle cells. Magnolol also elevated the HO-1 abundance in endothelial cells of healthy and remodeling veins (Fig. 7D).

## Discussion

Up to 70,000 plant species have been screened for their medicinal uses and of the new chemical entities covering the diseases between 1981 and 2002 up to 67% were estimated to be of natural, non-synthetic origins^27,28^. Extracts from the bark and flower of the tree of Magnolia officinalis as well as from other species of the Magnoliaceae family have been used in traditional medicine in the past and continue to be in use today in modern clinical practice in countries such as Japan^9,29–32^. Of the several neolignans identified, Magnolol was found to be the predominant bioactive constituent (0.78–7.65%) in at least two Magnoliaceae species^9,33–36^.

A number of prior studies report pleotropic effects of Magnolol on endothelial cells in culture in the context of exogenous induction with cytokines or growth factors^26,37–39^. Here for the first time we report on the functional impact of Magnolol on human endothelial cells subjected to biomechanical stretch as a surrogate for biomechanically stressed venous endothelium in small veins exposed to hypertension *in vivo*. Treatment of HUVECs with Magnolol resulted in a significant reduction in proliferation while the viability of these cells was not affected. Levels of phosphorylated ERK1/2 were reduced in HUVECs subjected to biomechanical stretch when pre-treated with Magnolol. Similarly, suppression of MAP kinase signalling (ERK, JNK and p38) and PI3K/AKT signalling pathways has been reported in mouse embryonic stem (mES)/embryoid body (EB)-derived endothelial-like cells treated with Magnolol as well as in TNF-α-induced human artery endothelial cell (HAEC) cultures (ERK, JNK and p38 kinase phosphorylation)^37,40^. In cardiac fibroblasts, Magnolol attenuated ERK phosphorylation in the context of ROS-mediated cardiac remodeling^41^. Indeed, based on structural characterization, Magnolol was identified as a suitable lead compound for development of antioxidants due to the hydroxyl group located on the biphenolic moiety which may provide the basics for a direct ROS scavenging effect^23,24^. Significant anti-oxidative effects of Magnolol were reported in HAECs at baseline and following TNFα stimulation^26^ while Magnolol (up to 5 µM) exerted limited anti-oxidative effects on HUVECs treated with hydrogen peroxide^25^. In light of these findings, we report a reduction in the level of superoxide anions in HUVECs treated with Magnolol under basal conditions while Magnolol had no effect on the enhanced superoxide production in HUVECs subjected to biomechanical stretch. Interestingly, both the depletion of ROS as well as the anti-proliferative effect of Magnolol appeared to be dependent on its induction of HO-1 expression rather than on its reported ROS scavenging effects.

An anti-angiogenic effect of Magnolol was also reported previously using a HUVEC tube formation assay, however, this effect was found to be hypoxia/VEGF-specific as Magnolol did not affect HUVEC angiogenesis under normoxic conditions^42^. In another study, Magnolol exhibited anti-angiogenic properties during VEGF-induced tube formation of HUVECs *in vitro* as well as VEGF-induced proliferation and chemotactic motility of these cells. These effects were thought to be mediated via VEGF-induced Ras activation as well inhibition of ERK, FAK, p38 and PI3K/Akt kinases and signalling pathways^13^. In the current study and based on employing a more sophisticated three dimensional angiogenesis assay, we show that Magnolol does not exhibit an effect on VEGF-induced angiogenesis in HUVECs and bears no harmful effect on the viability and functionality of these cells. Thus, it appears to be unlikely that transdermal administration of Magnolol interferes with wound healing processes or vascular maintenance which is also supported by our *in vivo* results. In fact, the potential for transdermal Magnolol administration has been explored in the context of skin ageing and UV-induced skin tumor formation^43–45^. Similarly, an anti-cancer activity of Magnolol has been previously reported, attributed primarily to the Magnolol-dependent reduction in MMP9 expression^46^.

Here for the first time we show that Magnolol effectively interferes with remodeling of small veins when applied as a topical cream formulation. In this context, we have previously shown that an increase in venous filling pressure is sufficient to drive venous remodeling in mice by stimulating venous endothelial and smooth muscle cells to activate the transcription factor AP-1, to produce matrix-metalloproteinases and to proliferate^18^. As superficial veins are connected to deeper venous plexus, they may thus be affected by an increased filling pressure in the underlying venous network, especially in the lower extremities. Assuming a direct connection, this biomechanical stimulus is likely to apply to both (deep) varicose and (superficial) spider vein development in humans. While remodeling of larger veins will involve architectural changes of the smooth muscle cell layers, remodeling of small superficial veins has to the most part be reliant on the endothelium - especially in the mouse veins which are only minimally covered with mural cells. Magnolol may thus have the capacity to inhibit the corresponding venous remodeling process on multiple levels by amplifying the HO-1-mediated resistance of endothelial cells to ROS-mediated proliferative stimuli and by preventing reconstruction of the venous wall by blocking the proteolytic activity upon biomechanical load.

Furthermore, considering the microarray data, Magnolol may provoke additional responses of vascular cells. For instance, it appears to influence the transcription of genes involved in the regulation of metabolic pathways in biomechanically stressed endothelial cells. In principle, an altered metabolic state has the capacity to control the activity of the endothelium^47^ and could therefore contribute to the inhibitory effects of Magnolol as observed in this study. Additionally, Magnolol stimulated the expression of C1QTNF3 a member of the C1q/TNF-related protein (CTRP) family that has been characterized as a structural and functional adiponectin paralog^48^. C1QTNF3 has been associated with low body mass indices^49^ and has the capacity to lower blood glucose levels in *ob/ob* mice^50^ and to attenuate high glucose-mediated pro-inflammatory activation of cultured human microvascular endothelial cells^51^. By triggering the expression of this adipokine in biomechanically stressed endothelial cells, Magnolol may thus indirectly alter their metabolic status and attenuate their overall level of activity.

Collectively, Magnolol should not be considered as a highly specific and unifunctional ligand that triggers one signaling pathway in a single cell type. In endothelial cells it may, however, bear pleiotropic effects which do not interfere with basic functions but may effectively counteract stress-induced responses.

## Electronic supplementary material

Supplements

## References

[1] Partsch H (2009). Varicose veins and chronic venous insufficiency. VASA Z. Gefasskrankheiten.

[2] Gloviczki P (2011). The care of patients with varicose veins and associated chronic venous diseases: clinical practice guidelines of the Society for Vascular Surgery and the American Venous Forum. J. Vasc. Surg..

[3] Allen, L. Assessment and management of patients with varicose veins. *Nurs*. *Stand*. *R*. *Coll*. *Nurs*. *G*. *B*. 1987 **23**, 49–57; quiz 58 (2009). 10.7748/ns2009.02.23.23.49.c6801 19263906

[4] Tisi, P. V. Varicose veins. B*MJ Clin*. *Evid*. **2011** (2011). PMC3217733 21477400

[5] Campbell B (2006). Varicose veins and their management. BMJ.

[6] Sisto T (1995). Prevalence and risk factors of varicose veins in lower extremities: mini-Finland health survey. Eur. J. Surg. Acta Chir..

[7] Pfisterer L, König G, Hecker M, Korff T (2014). Pathogenesis of varicose veins - lessons from biomechanics. VASA Z. Gefasskrankheiten.

[8] Atta, H. M. Varicose Veins: Role of Mechanotransduction of VenousHypertension. *Int*. *J*. *Vasc*. *Med*. **2012** (2012). 10.1155/2012/538627 PMC3303599 22489273

[9] Lee Y-J (2011). Therapeutic applications of compounds in the Magnolia family. Pharmacol. Ther..

[10] Chen YL (2001). Magnolol, a potent antioxidant from Magnolia officinalis, attenuates intimal thickening and MCP-1 expression after balloon injury of the aorta in cholesterol-fed rabbits. Basic Res. Cardiol..

[11] Karki R, Ho O-M, Kim D-W (2013). Magnolol attenuates neointima formation by inducing cell cycle arrest via inhibition of ERK1/2 and NF-kappaB activation in vascular smooth muscle cells. Biochim. Biophys. Acta.

[12] Mukherjee PK, Maity N, Nema NK, Sarkar BK (2011). Bioactive compounds from natural resources against skin aging. Phytomedicine Int. J. Phytother. Phytopharm..

[13] Kim KM (2013). Magnolol suppresses vascular endothelial growth factor-induced angiogenesis by inhibiting Ras-dependent mitogen-activated protein kinase and phosphatidylinositol 3-kinase/Akt signaling pathways. Nutr. Cancer.

[14] Gu Y, Lewis DF, Zhang Y, Groome LJ, Wang Y (2006). Increased Superoxide Generation and Decreased Stress Protein Hsp90 Expression in Human Umbilical Cord Vein Endothelial Cells (HUVECs) from Pregnancies Complicated by Preeclampsia. Hypertens. Pregnancy Off. J. Int. Soc. Study Hypertens. Pregnancy.

[15] Wen, Y.-D. *et al*. Hydrogen Sulfide Protects HUVECs against Hydrogen Peroxide Induced Mitochondrial Dysfunction and Oxidative Stress. *PLoS ONE* **8** (2013). 10.1371/journal.pone.0053147 PMC3564806 23393548

[16] Heiss M (2015). Endothelial cell spheroids as a versatile tool to study angiogenesis *in vitro*. FASEB J..

[17] North KA, Sanders AG (1958). The development of collateral circulation in the mouse’s ear. Circ. Res..

[18] Feldner A, Otto H, Rewerk S, Hecker M, Korff T (2011). Experimental hypertension triggers varicosis-like maladaptive venous remodeling through activator protein-1. FASEB J. Off. Publ. Fed. Am. Soc. Exp. Biol..

[19] Jufri NF, Mohamedali A, Avolio A, Baker MS (2015). Mechanical stretch: physiological and pathological implications for human vascular endothelial cells. Vasc. Cell.

[20] Zheng W, Christensen LP, Tomanek RJ (2008). Differential effects of cyclic and static stretch on coronary microvascular endothelial cell receptors and vasculogenic/angiogenic responses. Am. J. Physiol. Heart Circ. Physiol..

[21] Anwar MA, Shalhoub J, Lim CS, Gohel MS, Davies AH (2012). The effect of pressure-induced mechanical stretch on vascular wall differential gene expression. J. Vasc. Res..

[22] Eschrich, J. *et al*. Varicose Remodeling of Veins Is Suppressed by 3-Hydroxy-3-Methylglutaryl Coenzyme A Reductase Inhibitors. *J*. *Am*. *Heart Assoc*. **5** (2016). 10.1161/JAHA.115.002405 PMC4802467 26908399

[23] Ho JH-C, Hong C-Y (2012). Cardiovascular protection of magnolol: cell-type specificity and dose-related effects. J. Biomed. Sci..

[24] Wang Y, Li C-Y, Lin I-H, Lee A-R, Hu M-K (2002). Synthesis and radical scavenging of novel magnolol derivatives. J. Pharm. Pharmacol..

[25] Bertin R (2016). Activity of myricetin and other plant-derived polyhydroxyl compounds in human LDL and human vascular endothelial cells against oxidative stress. Biomed. Pharmacother. Biomedecine Pharmacother..

[26] Chen Y-H, Lin S-J, Chen J-W, Ku H-H, Chen Y-L (2002). Magnolol attenuates VCAM-1 expression *in vitro* in TNF-alpha-treated human aortic endothelial cells and *in vivo* in the aorta of cholesterol-fed rabbits. Br. J. Pharmacol..

[27] Veeresham P, (Dr) C (2012). Natural products derived from plants as a source of drugs. J. Adv. Pharm. Technol. Res..

[28] Newman DJ, Cragg GM, Snader KM (2003). Natural products as sources of new drugs over the period 1981–2002. J. Nat. Prod..

[29] Luo L, Nong Wang J, Kong LD, Jiang QG, Tan RX (2000). Antidepressant effects of Banxia Houpu decoction, a traditional Chinese medicinal empirical formula. J. Ethnopharmacol..

[30] Hsu, H. & Hsü, C. *Commonly Used Chinese Herb Formulas with Illustrations*. (Oriental Healing Arts Institute, 1980).

[31] Fukushima M (1997). [Profiles of effects of traditional oriental herbal medicines on central nervous systems in humans–assessment of saiboku-to and saiko-ka-ryukotsu-borei-to using EEG and pharmacokinetics of herbal medicine-derived ingredients as indices]. Seishin Shinkeigaku Zasshi.

[32] Iwasaki K (2000). The effects of the traditional chinese medicine, ‘Banxia Houpo Tang (Hange-Koboku To)’ on the swallowing reflex in Parkinson’s disease. Phytomedicine Int. J. Phytother. Phytopharm..

[33] Choi NH (2009). Effects of neolignans from the stem bark of Magnolia obovata on plant pathogenic fungi. J. Appl. Microbiol..

[34] Matsuda H (2001). Effects of constituents from the bark of Magnolia obovata on nitric oxide production in lipopolysaccharide-activated macrophages. Chem. Pharm. Bull. (Tokyo).

[35] Lee YK (2009). Protective effect of the ethanol extract of Magnolia officinalis and 4-O-methylhonokiol on scopolamine-induced memory impairment and the inhibition of acetylcholinesterase activity. J. Nat. Med..

[36] Lee JW (2010). Inhibitory effect of ethanol extract of Magnolia officinalis and 4-O-methylhonokiol on memory impairment and neuronal toxicity induced by beta-amyloid. Pharmacol. Biochem. Behav..

[37] Liang C-J (2014). Magnolol reduced TNF-α-induced vascular cell adhesion molecule-1 expression in endothelial cells via JNK/p38 and NF-κB signaling pathways. Am. J. Chin. Med..

[38] Liang X (2015). Magnolol administration in normotensive young spontaneously hypertensive rats postpones the development of hypertension: role of increased PPAR gamma, reduced TRB3 and resultant alleviative vascular insulin resistance. PloS One.

[39] Chen S-C, Chang Y-L, Wang DL, Cheng J-J (2006). Herbal remedy magnolol suppresses IL-6-induced STAT3 activation and gene expression in endothelial cells. Br. J. Pharmacol..

[40] Kim GD, Oh J, Park H-J, Bae K, Lee SK (2013). Magnolol inhibits angiogenesis by regulating ROS-mediated apoptosis and the PI3K/AKT/mTOR signaling pathway in mES/EB-derived endothelial-like cells. Int. J. Oncol..

[41] Liou J-Y (2009). Magnolol depresses urotensin-II-induced cell proliferation in rat cardiac fibroblasts. Clin. Exp. Pharmacol. Physiol..

[42] Chen M-C, Lee C-F, Huang W-H, Chou T-C (2013). Magnolol suppresses hypoxia-induced angiogenesis via inhibition of HIF-1α/VEGF signaling pathway in human bladder cancer cells. Biochem. Pharmacol..

[43] Shen J-L (2010). Honokiol and magnolol as multifunctional antioxidative molecules for dermatologic disorders. Mol. Basel Switz..

[44] Chilampalli C (2011). Effects of magnolol on UVB-induced skin cancer development in mice and its possible mechanism of action. BMC Cancer.

[45] Im A-R, Song JH, Lee MY, Chae S (2015). Magnolol reduces UVB-induced photodamage by regulating matrix metalloproteinase activity. Environ. Toxicol. Pharmacol..

[46] Liu, Y. *et al*. The natural compound magnolol inhibits invasion and exhibits potential in human breast cancer therapy. *Sci*. *Rep*. **3** (2013). 10.1038/srep03098 PMC3827615 24226295

[47] De Bock K, Georgiadou M, Carmeliet P (2013). Role of endothelial cell metabolism in vessel sprouting. Cell Metab..

[48] Wong GW, Wang J, Hug C, Tsao T-S, Lodish HF (2004). A family of Acrp30/adiponectin structural and functional paralogs. Proc. Natl. Acad. Sci. USA.

[49] Wolf RM (2015). Lower Circulating C1q/TNF-Related Protein-3 (CTRP3) Levels Are Associated with Obesity: A Cross-Sectional Study. PloS One.

[50] Peterson JM, Wei Z, Wong GW (2010). C1q/TNF-related protein-3 (CTRP3), a novel adipokine that regulates hepatic glucose output. J. Biol. Chem..

[51] Yan Z (2017). CTRP3 is a novel biomarker for diabetic retinopathy and inhibits HGHL-induced VCAM-1 expression in an AMPK-dependent manner. PloS One.

