# Supplementary material for: Magnolol inhibits venous remodeling in mice
Source: Sci Rep. 2017 Dec 19;7:17820. doi: 10.1038/s41598-017-17910-0 (PMC5736655; doi:10.1038/s41598-017-17910-0)
Supplement: Supplementary file 1 — Supplements [file 41598_2017_17910_MOESM1_ESM.pdf]

SUPPLEMENT

Magnolol inhibits venous remodeling in mice

Hanna Kuk[1], M.Sc., Caroline Arnold[1], PhD, Ralph Meyer[1], M.Sc., Markus Hecker[1], PhD and Thomas Korff[1], PhD

[1]Institute of Physiology and Pathophysiology, Division of Cardiovascular Physiology, University of Heidelberg

Address for correspondence:
Thomas Korff, PhD
University of Heidelberg
Institute of Physiology and Pathophysiology
Division of Cardiovascular Physiology
Im Neuenheimer Feld 326
69120 Heidelberg, Germany
Phone: 49-6221-544131
FAX: 49-6221-544038
E-mail: korff@physiologie.uni-heidelberg.de

SUPPLEMENT

Suppl. Figure 1

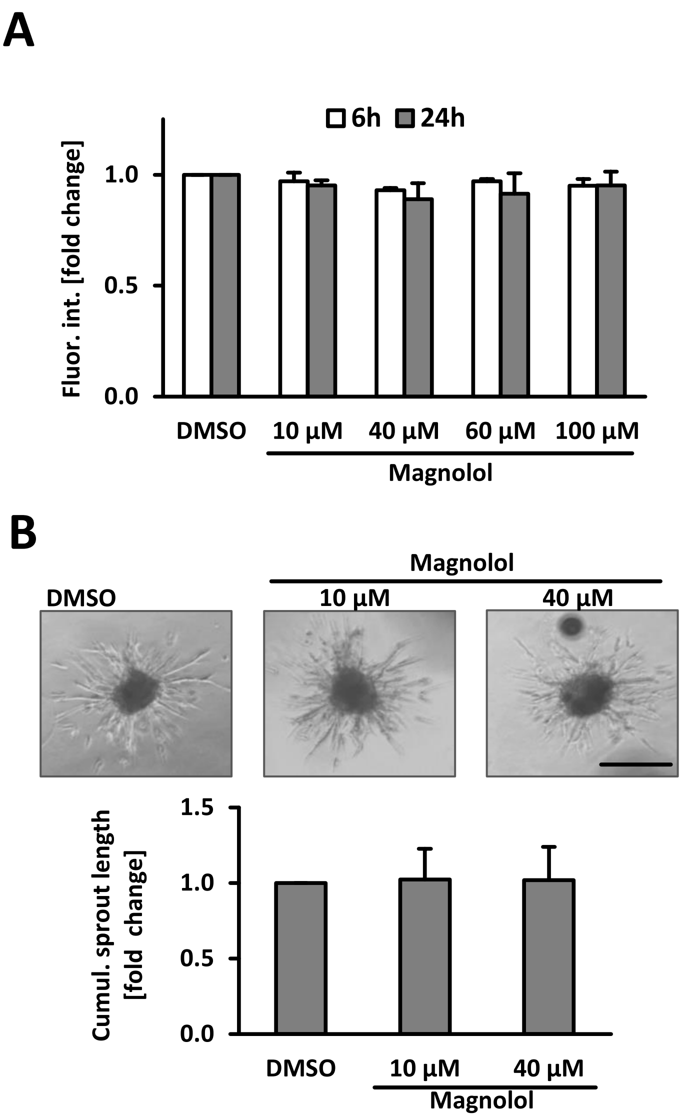

Suppl. Figure 1. Magnolol does not affect cell viability or angiogenic response (A) HUVECs were treated with the indicated concentrations of Magnolol for 6 and 24 hours and cell viability was determined by the PrestoBlue cell viability assay. The fluorescence intensity was normalized to DMSO vehicle treated control. No significant decrease in cell viability has been observed (n=3). **(B)** HUVEC spheroids were embedded in a collagen matrix supplemented with VEGF (25 ng/mL) and treated with Magnolol or DMSO vehicle control. No significant decrease in cell viability has been observed (n=3, scale bar: 100 µm).

SUPPLEMENT

Suppl. Figure 2

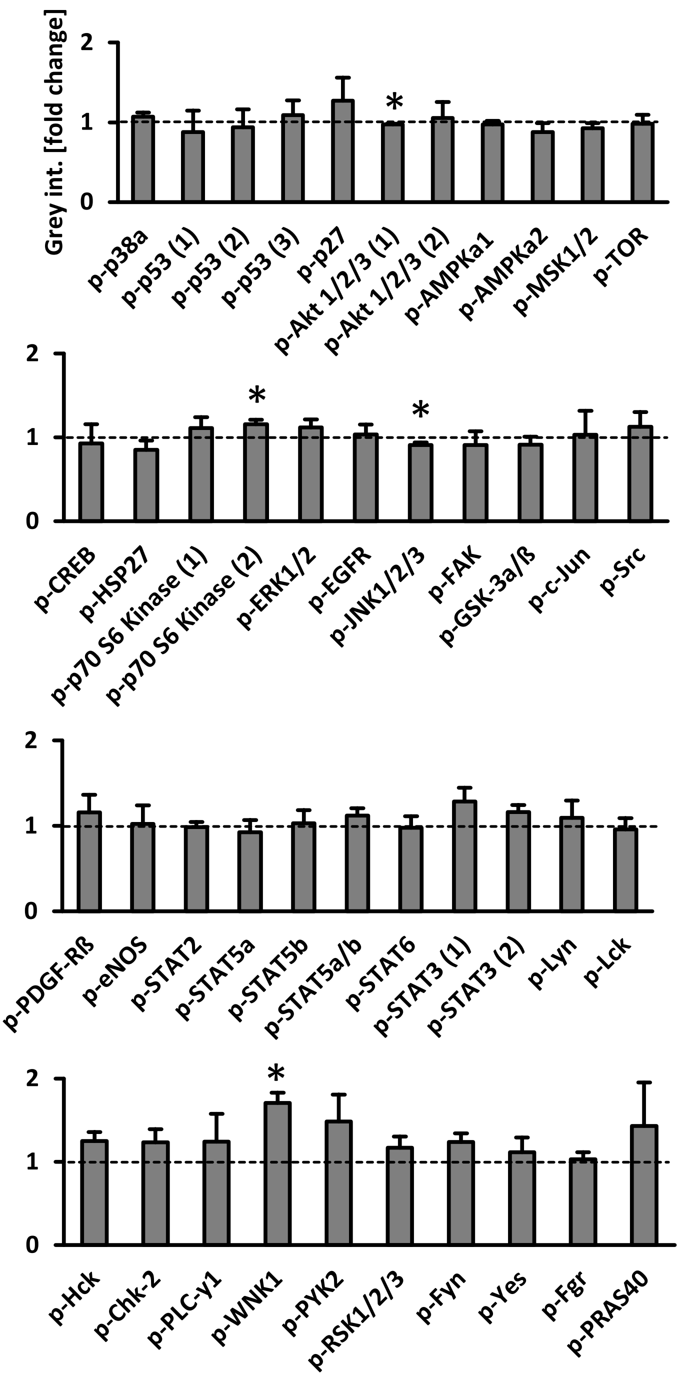

Suppl. Figure 2. Profile of phosphorylation levels of various signaling upon Magnolol treatment. HUVECs were pre-treated with Magnolol (40 µM) or DMSO vehicle control for 1.5 hours. Profiling of proteins involved in crucial signaling cascades was performed on cell lysates to analyze their relative phosphorylation of phosphokinases and their substrates (all data are shown as compared to the DMSO static control which was set to 1 (dotted line), *$p<0.05$ vs. static control, n=3)

The following phosphorylation sites were assessed (in alphabetical order): Akt 1/2/3 (1: S473; 2: T308), AMPK alpha1 (T183), AMPK alpha2 (T172), beta-Catenin (global), Chk-2 (T68), c-Jun (S63), CREB (S133), EGF R (Y1086), eNOS (S1177), ERK1/2 (T202/Y204, T185/Y187), FAK (Y397), Fgr (Y412), Fyn (Y420), GSK-3 alpha/beta (S21/S9), Hck (Y411), HSP27 (S78/S82), HSP60, JNK 1/2/3 (T183/Y185, T221/Y223), Lck (Y394), Lyn (Y397), MSK1/2 (S376/S360), p27 (T198), p38 alpha (T180/Y182), p53(1: S392; 2: S46; 3: S15), p70 S6 Kinase (1:T389; 2: T421/S424), PDGF R beta (Y751), PLC gamma-1 (Y783), PRAS40 (T246), Pyk2 (Y402), RSK1/2/3 (S380), Src (Y419), STAT2 (Y689), STAT3 (1: Y705; 2: S727), STAT5a (Y699), STAT5a/b (Y699), STAT5b (Y699), STAT6 (Y641), TOR (S2448), WNK-1 (T60), Yes (Y426).

Suppl. Figure 3

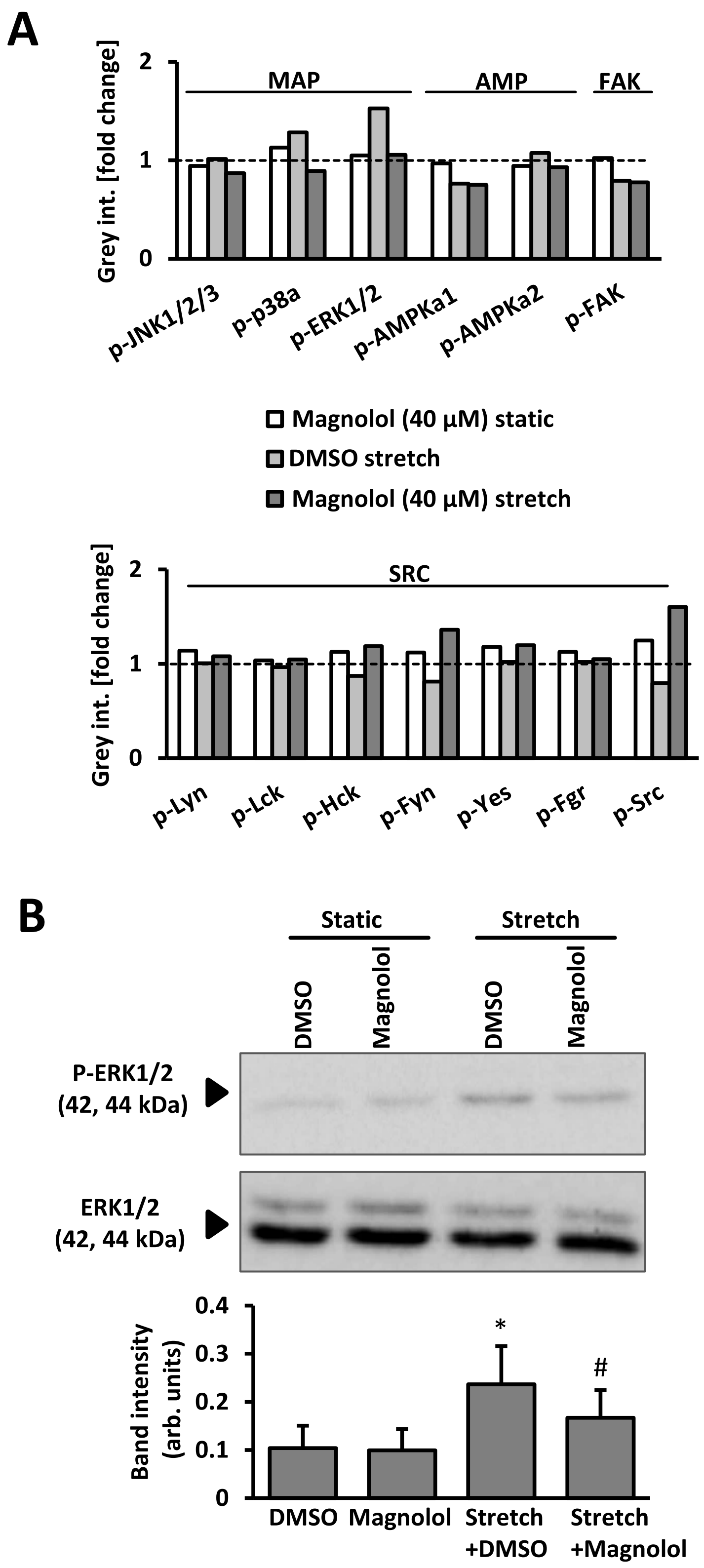

Suppl. Figure 3. Phosphorylation profile of kinases upon Magnolol treatment. (A) HUVECs were pre-treated with Magnolol (40 µM) or DMSO vehicle control for 1.5 hours and subject to biomechanical stretch for 15 min. Cell lysates were processed to exemplarily analyze the relative phosphorylation of MAP- and Src-kinases (regulators of signal transduction and cellular activity), AMP kinases (regulators of metabolism) and FAK (regulators of cell adhesion and motility) are shown as compared to the DMSO static control (set to 1; dotted line). **(B)** Western blot of p-ERK1/2 levels of HUVECs subject to 15 min of stretch with or without Magnolol treatment (*p<0.05 DMSO static vs. DMSO stretch; #p<0.05 DMSO stretch vs. Magnolol stretch, n=5).

SUPPLEMENT

Suppl. Figure 4

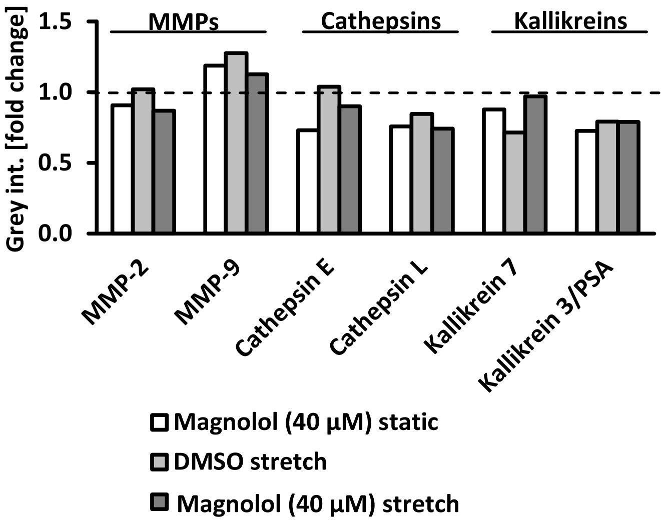

Suppl. Figure 4. Magnolol bears minimal effect on the expression of proteases. (A) HUVECs were exposed to biomechanical stretch for 24 hours with or without Magnolol pretreatment (40 µM). Changes in the relative protein level of proteases were screened and presented as compared to the DMSO static control (set to 1, dotted line).

SUPPLEMENT

Suppl. Figure 5

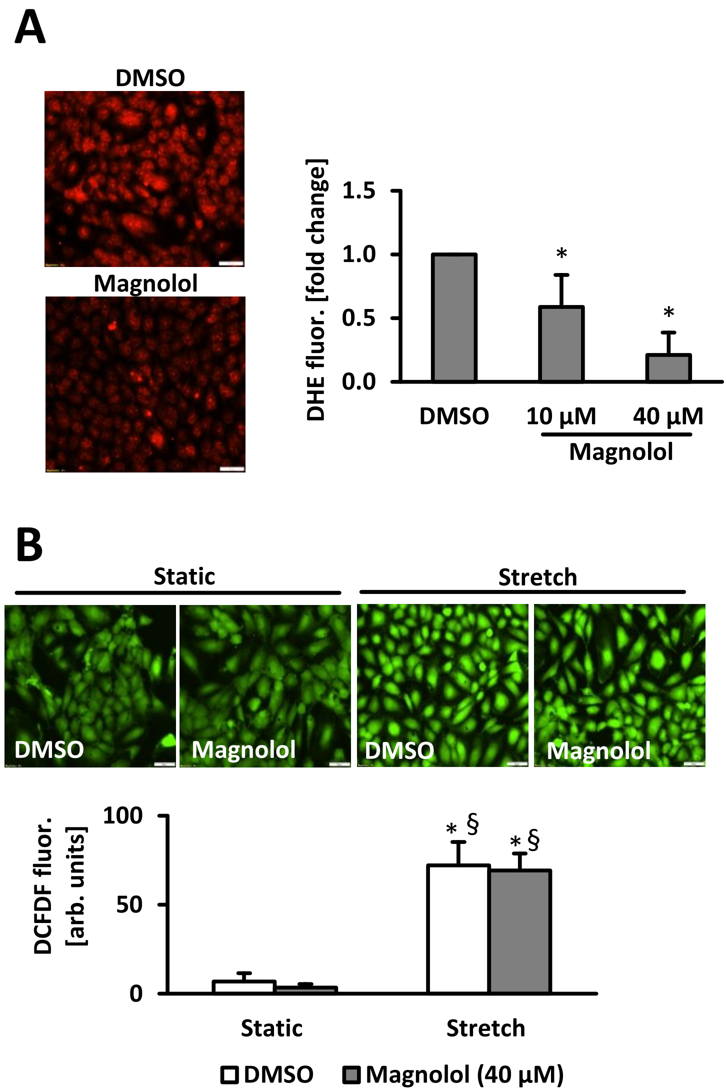

Suppl. Figure 5. Magnolol attenuates endogenous ROS levels in HUVECs but not upon stretch. HUVECs were treated with Magnolol (40 µM) or DMSO control vehicle for 24 hours **(A)** followed by DHE staining and quantitative immuno-fluorescence analyses to assess the levels of superoxide production (red fluorescence, *p<0.05 vs. DMSO control; n=6-7, scale bars = 50 µm). **(B)** HUVECs were pre-treated with Magnolol or DMSO control vehicle for 1.5 hours and subject to 24 hours of biomechanical stretch. DCFDF staining was carried out to assess the levels of both superoxide and peroxide (C, *p <0.05 vs. DMSO static, §p<0.05 vs. Magnolol static, bars represent the mean ±SD of fluorescence values obtained from 6-7 microscopic fields of view of 1 out of 3 experiments with comparable results, scale bar: 50 µm).

SUPPLEMENT

Suppl. Figure 6

GENE NAME	Regulation (log2-fold)	p-value ANOVA
C1QTNF3	0.92	0.0384
IGHV3-15	0.60	0.0030
HMOX1	0.53	0.0126
MIR539	-0.50	0.0293
**SIGNALING PATHWAY**	**Regulation (log2-fold)**	**p-value ANOVA**
Ovarian steroidogenesis	1.63	0.006
Ascorbate and aldarate metabolism	1.59	0.019
Starch and sucrose metabolism	1.55	0.012
Nitrogen metabolism	-1.98	0.0000

Suppl. Figure 6. Magnolol significantly up-regulated HMOX-1 mRNA expression in HUVECs upon stretch. HUVECs were pre-treated with DMSO or Magnolol (40 µM) for 1.5 hours and subject to 6h of biomechanical stretch. Microarray procedure was carried out on the extracted total RNA from each sample set. Genes and signaling pathways which were significantly up- (green) or down (red) –regulated in the Magnolol group upon stretch as compared to the DMSO control group upon stretch (Selection criteria: at least 0.5-fold(log2) for individual genes and at least 1.5-fold(log2) for pathway analysis, n=3).
